# From collective memory to clinical cases: analyzing political delusions in patients with psychotic disorders

**DOI:** 10.1186/s12888-025-07518-4

**Published:** 2025-11-03

**Authors:** Ahmet Selim Başaran, Hande Gazey, Sena Çağlayan, Selçuk Candansayar

**Affiliations:** 1 Department of Psychiatry, Unye State Hospital, Ordu, Turkey; 2 Department of Psychiatry, Bakırköy Mazhar Osman Mental Health and Neurological Diseases Education and Research Hospital, İstanbul, Turkey; 3 Department of Psychiatry, Ilgın Dr. Vefa Tanır State Hospital, Konya, Turkey; 4 Department of Psychiatry, Faculty of Medicine, Gazi University, Ankara, Turkey

**Keywords:** Political delusions, Psychotic disorders, Delusional content, Socio-political context, Thematic analysis, Qualitative research, Phenomenology, Turkey

## Abstract

**Background:**

Politically charged public life supplies powerful symbols that can be recruited into delusional meaning-making. This study examined how political contexts shape the content and experiential structure of delusions in patients with psychotic disorders in Turkey.

**Methods:**

We conducted a retrospective, archive-based qualitative study of inpatient psychiatric records from a tertiary hospital spanning 1985–2024. Of 1,657 records screened by two clinicians, 122 cases with sufficiently rich, politically themed delusional content were included following consensus review. Analysis used reflexive thematic analysis in an inductive, primarily semantic mode. All narratives were analyzed in Turkish to preserve nuance. Descriptive statistics summarized sample characteristics; no inferential tests were performed.

**Results:**

The sample comprised 122 inpatients (mean age 36.57 years; 68.9% male). Diagnoses were schizophrenia (66.4%), schizoaffective disorder (13.1%), delusional disorder (10.7%), and brief psychotic disorder (9.8%). These themes were grouped into two main categories: experiences of a persecutory political world, and the reorganization of the self through political narratives. Eight themes captured recurring constellations of actors, plots, and experiential disruptions: (1) an intrusive state and collapse of ontological distance, with surveillance and mind–body control motifs (39.5%); (2) grandiosity as compensatory self-organization via identification with national or global leaders (13.7%); (3) persecutory threat based on ideological identity (12.1%); (4) the foreign state as enemy, extending persecution to a transnational horizon (10.5%); (5) the personalized leader’s gaze transforming symbolic authority into direct persecution (8.1%); (6) ethno-religious others as ontological contaminants (6.5%); (7) “psychotic nationalism,” merging self and nation amid catastrophic scenarios (3.2%); and (8) illegitimate pursuers, such as terrorist or nameless clandestine groups (6.5%). Across themes, patients’ narratives reflected blurred self–world boundaries, externalized agency, and the recruitment of widely circulated political symbols.

**Conclusions:**

Politically themed delusions are structured, culturally anchored narratives rather than incidental noise. In the Turkish context, state institutions, charismatic leaders, foreign powers, clandestine organizations, and ethno-religious figures recurrently organize delusional meaning. Context-attentive assessment and formulation—mapping each patient’s political horizon of meaning, anticipating risk around salient public events, and integrating proportionate psychoeducation—may improve engagement and safety. Prospective, mixed-methods studies linking first-person accounts with timelines of public events are warranted to clarify temporal coupling and guide interventions.

**Clinical trial number:**

Not applicable.

## Background

Delusions are one of the core clinical symptoms that are diagnostic of almost all psychotic disorders. The phenomenological examination of delusions has continued to be of clinical interest since the 19th century [1, 2]. This is related to the idea that delusions serve as a tool for understanding the patient’s inner world [3, 4]. Additionally, studies indicate that there is also a relationship between the content of delusions and the world the patient inhabits [5–7]. However, with the development of biological psychiatry, there appears to have been a temporary decline in the focus on delusional content [8, 9]. Today, the challenges in classifying psychotic disorders have reached an impasse, leading to discussions about the necessity of returning to the concept of psychosis as an overarching framework [10]. This overarching concept suggests that a phenomenological examination of delusions, one of the core symptoms of psychosis, could also be beneficial for understanding the etiopathogenesis of psychotic disorders. One of the most important findings supporting this phenomenon is that the content of delusions in patients with schizophrenia changes over time [5]. Delusions evolve in response to new technological developments [11, 12], political processes [13, 14], sociocultural changes [15, 16], and popular music trends [17]. The change in delusional content influenced by history, culture, and politics also raises the question of whether the difference between psychotic and non-psychotic thought is quantitative or qualitative.

Phenomenological examinations of delusions have typically revealed classifications such as persecution, grandeur, jealousy, and religious delusions, which are often used in clinical practice [5, 18]. While this classification provides some insight into delusional content, it is not sufficient to fully understand the patient’s subjective experience. However, it is known that the frequency of these basic delusion classes varies according to political, social, or cultural changes [7, 16, 19]. For example, a study conducted in Slovenia showed that the frequency of persecution delusions increased, particularly among individuals with dissenting views, following Yugoslavia’s transition from a monarchical regime to communism [19].

Politics can be defined as the sum of activities related to decision-making processes and power relations among various groups, individuals, or organizations within society [20]. However, one of the definitions that can be used to explain politics is based on Nancy Fraser’s triad. According to this, politics can be defined as a multidimensional process aimed at achieving justice through addressing economic inequalities (redistribution), recognizing identities and cultural differences (recognition), and ensuring individuals’ participation in democratic processes and equality in political representation (representation) [21]. It is likely that, as members of the society they live in, both the lives and thought content of patients with psychotic disorders are influenced by the political environment they find themselves in. This thought content may take on a delusional nature in patients who have not reached complete remission or are in an active phase of the illness.

As far as we know, there is no qualitative study specifically on delusions with political content, but a few studies can offer insights into this topic. One was conducted on Egyptian patients in 1976 [22]. The delusional content provided as examples in this study reflects the political tensions of the time, being related to the conflicts between Arabs and Israelis. In a study by Kim and colleagues in 1993, it was noted that patients’ delusions related to politics were shaped by their country and political agenda [23]. According to the data from these studies, most politically themed delusions are classified as persecution or grandiosity delusions [24]. Additionally, many politically-themed delusions also resemble conspiracy theories [25]. These conspiracies typically involve authorities such as politicians, leaders, and scientists or socially marginalized groups like Jews, Muslims, and Black people.

Turkey’s recent history is a highly complex period marked by successive major economic, political, and cultural crises, and the records of the patients included in this study were documented during these periods. Many of these patients were born, grew up, and lived during a time marked by the Cold War, with its global impacts, the right-left conflicts that engulfed the country, military coups, elections, and economic issues [26]. This study will carry out a thematic analysis of political themes in the delusions of patients with psychotic disorders in Turkey. The aim is to reveal the relationship between political events and the content of delusions, addressing a gap in the literature. While there are a limited number of studies on politically themed delusions, these studies mostly highlight persecution and grandiosity delusions (Kim et al., 1993; El Sendiony, 1976). However, the dynamics of political delusions and their relationship to historical-political events have not yet been thoroughly explored. In this context, understanding the content of politically themed delusions in the light of historical-political events in Turkey and the world can help us assess individual pathology and better understand the socio-political environment in which these individuals live.

## Methods

### Study design

This retrospective, archive-based qualitative study employed reflexive thematic analysis as outlined by Braun and Clarke to identify and organize recurring patterns of meaning in politically themed delusions documented in inpatient psychiatric records [27]. A qualitative design was selected to capture the context-sensitive and interpretive aspects of delusional meaning-making while maintaining a transparent, auditable analytic pathway focused on patterned, recurrent meanings rather than isolated anecdotes. This study was reported in accordance with the Standards for Reporting Qualitative Research (SRQR) checklist [28].

### Setting and ethical considerations

The study drew on archived inpatient records from the Psychiatry Department of Gazi University Hospital, a tertiary-level institution in Turkey. The dataset spanned 1985–2024, encompassing documentation generated under multiple diagnostic systems from DSM-III-R through DSM-5. Ethical approval was granted by the University’s Research Ethics Committee (Approval No: 2024 − 1481, Date: 24.09.2024), and the hospital administration provided formal permission to access records. In accordance with the Helsinki Declaration, all data were anonymized before analysis, and no personally identifiable information was extracted or reported.

### Inclusion and exclusion criteria

Eligible cases were adult inpatients (18–65 years) with a documented diagnosis of schizophrenia spectrum or other psychotic disorders during the study period. Diagnoses recorded under earlier DSM editions were harmonized to current nosological boundaries by a senior psychiatrist (SC). Exclusion criteria were: a primary diagnosis of a non-psychotic disorder (e.g., major depressive disorder, dementia), the presence of a neurological condition likely to affect cognition (e.g., epilepsy, brain tumors), and insufficient narrative material to assess delusional content.

### Data retrieval and operationalization of political delusions

A total of 1,657 patient records were screened independently by two researchers trained in clinical psychiatry and qualitative methods. Records included admission notes, discharge summaries, daily progress reports, and mental status examinations written by attending psychiatrists. Politically themed delusions were operationally defined as persistent false beliefs centrally involving state institutions, political figures, ideological groups, or political events situated within national or international contexts, accompanied by high subjective conviction and clear clinical salience. Cases were included only when both reviewers independently judged the delusional content to be politically central and sufficiently rich for analysis; disagreements were resolved in consensus meetings with the senior authors, resulting in a final analytic cohort of 122 patients.

### Analytic procedures

Analysis followed the six-phase reflexive thematic analysis model in an inductive and primarily semantic mode [27]. During familiarization, the team collated all eligible narrative material for each case and engaged in repeated close readings to achieve immersion. Analysts produced initial memos documenting recurring actors, actions, settings, and salient political referents (e.g., named institutions, historical markers) to support later theme construction.

In initial coding, two analysts independently conducted line-by-line or segment-based coding across the corpus, assigning concise labels to meaningful units (for example, surveillance by the state, direct address by a leader, foreign power intervention, ideological identity as target). Coding was data-driven; a provisional codebook was iteratively developed with explicit definitions, inclusion/exclusion criteria, and illustrative extracts, while reflexive memos captured decision points such as splitting or collapsing codes.

Theme construction proceeded by clustering codes around central organizing concepts to form candidate themes. Constant comparison was used to test whether clusters represented patterned meanings across multiple cases rather than idiosyncratic features. The team developed thematic maps and code–theme matrices to articulate relationships among themes and potential subthemes.

During theme review and refinement, coherence was assessed at two levels: within-theme coherence of the collated extracts and fit of the candidate thematic map to the entire dataset. Where necessary, themes were split, merged, or re-scoped to reduce overlap and enhance parsimony without sacrificing coverage of the corpus.

In defining and naming themes, detailed analytic definitions were written for each retained theme, specifying the central organizing concept, typical and variant features, and clear boundaries, accompanied by representative extracts. Concise, communicative names were assigned to reflect each theme’s focus and scope, and the finalized codebook recorded definitions and naming rationales.

Finally, in producing the analytic account, the team selected vivid, representative quotations to evidence each theme and crafted a narrative demonstrating how the themes addressed the study aim. External theoretical frameworks were not imported as coding criteria; instead, claims were warranted by direct linkage to the archived narratives, and, where appropriate, statements about theme prevalence within the dataset were provided to enhance transparency. Analysts met regularly to compare coding, discuss boundary cases, and update the codebook. Differences were treated as an analytic resource and resolved through discussion-based consensus, with all decisions recorded in an audit trail including date-stamped memos, codebook versions, and thematic maps. All materials were stored in a structured project repository to support transparency and dependability. All analysis was conducted in the original Turkish to preserve cultural and semantic nuance. Translation of theme names and illustrative quotations into English was performed by bilingual team members and reviewed by a second bilingual author to ensure conceptual accuracy and semantic equivalence.

### Reflexivity

The research team consists of psychiatrists actively practicing in Turkey. This shared socio-political context provided an insider perspective, facilitating the recognition of culturally and historically specific references within patient narratives (e.g., specific political figures, events, and organizations). However, we were mindful that our own experiences and perspectives could shape the interpretation of the data. To mitigate potential bias, we engaged in regular peer debriefing sessions where we critically examined our coding decisions and interpretive assumptions. Disagreements were treated as opportunities for deeper reflection on the data, ensuring that the final themes were grounded in the participants’ documented experiences rather than our preconceptions.

## Results

This study investigated the phenomenological architecture and sociohistorical embedding of politically themed delusions in a clinical sample of 122 inpatients diagnosed with psychotic disorders. The patients had a mean age of 36.57 years, with 68.9% identified as male and 31.1% as female. Diagnostic distribution included schizophrenia (66.4%), schizoaffective disorder (13.1%), delusional disorder (10.7%), and brief psychotic disorder (9.8%) (Table 1). The delusions were not treated as isolated cognitive anomalies, but as meaningful reorganizations of the self–world relationship, shaped by the intersection of subjective vulnerability and political-symbolic imaginaries. The eight thematic clusters that emerged from the analysis were organized into two primary categories to better illustrate the core dynamics observed: experiences of a persecutory political world, and the reorganization of the self through political narratives (Table 2).

**Table 1:** Participant characteristics

		Sample *N* = 122	
		N	%
Sex	Female	38	31.1
	Male	84	68.9
Diagnoses	Schizophrenia	81	66.4
	Delusional Disorder	13	10.7
	Schizoaffective Disorder	16	13.1
	Brief Psychotic Disorder	12	9.8
**Age**		**Mean**	**S.D.**
		36.57	11.48

**Table 2:** The themes generated from the thematic analysis

Thematic category	Themes	Agent / subject	Core content	*n*	%
The Persecutory Political World	The Intrusive State and the Collapse of Ontological Distance	State institutions	Surveillance, tailing, mind/body control via “chips”; a politically intrusive Other.	49	39.5
	The Political Self Under Siege: Persecutory Threats Based on Ideological Identity	Opposing ideological camps	Identity-based tracking/punishment; hyper-meaning attributed to cues	15	12.1
	The Foreign Other as Enemy: Global Paranoia and Psychotic Cosmopolitics	Foreign states/powers	Cross-border intervention via satellites, agents, religious/economic mechanisms	13	10.5
	The Leader’s Gaze: From Symbolic Authority to Personalized Persecution	Named political leaders	Omniscient, all-encompassing leader as personal persecutor; personalized plots and threats.	10	8.1
	Illegitimate Pursuers: Terrorist Groups and Nameless Powers	Terrorist organizations	Persistent tracking/marking; mistrust of healthcare staff and attribution shifts.	8	6.5
The Self Reconfigured Through Political Myths	Grandiosity as a Response to Fragmentation: Historical Identification and Mythic Selves	The “self” identified with leaders	Intense identification with national/global figures; narratives of mission, destiny, and salvation.	17	13.7
	Contaminated Selves: The Ethno-Religious Other as Ontological Threat	Ethno-religious Others or cultural figures	Soul “being eaten,” poisoning, penetration into body/soul.	8	6.5
	Psychotic Nationalism: Threats to National Integrity and Self–State Mergers	The “nation/state” and enemy forces	Bombs under cities, industrial sabotage; the nation’s fate = the self’s fate.	4	3.2

### Category I: The persecutory political world

This category includes themes where the political environment is experienced as a source of direct, invasive, and personalized threat, leading to a collapse of boundaries between the self and powerful external entities.

#### The intrusive state and the collapse of ontological distance

This most common theme captures the collapse of ontological distance between the individual and public authority. Patients do not experience the state as an impersonal, external structure; instead, the police, military, or MIT (National Intelligence Organization) appear as intrusive entities penetrating thought, body, and space. Delusions often emerge as surveillance, being followed, and bodily manipulation—phenomena of passivity such as thought insertion, being controlled, or cognitive governance via “chips,” despite the absence of any clear political activity.

The state is watching everything. I know they implanted a chip in my brain, probably when I was last in the hospital, so they can control my thoughts and stop me from speaking out.

Everywhere I go, I see the same unmarked car. They are agents from the intelligence service, and they are just waiting for the right moment to arrest me. They think I know too much.

Structurally, the picture is an indication of self-dissolution: diminished affectivity and the patient’s obsessive attention to micro-events—glances, headlines, environmental sounds—which now become saturated with hostile meanings. The boundary between self and world becomes permeable; the state is transformed into a persecutory quasi-subject who sees, judges, and acts through the patient. Disturbances in body schema and interoception further somatize political fear (“devices under the skin,” “voices coming through the teeth”) and reinforce the sense of inner occupation. The world loses its intelligibility; agency is externally attributed to an all-powerful political Other.

#### The political self under siege: persecutory threats based on ideological identity

This theme captures a persecutory structure in which political identity—leftist, right-wing, Kemalist—ceases to be an abstract stance and becomes the very essence of the self targeted for punishment. Patients report that institutions or “opposing camps” (e.g., CIA, counter-guerilla, MIT) target not just their thoughts, but who they are. Ideology becomes hyper-corporeal: experienced as etched into the body and life history of the individual, transforming the social realm into a medium where one is exposed, judged, and condemned. Hyper-reflexive scanning of headlines, gestures, and online posts becomes the interpretation of targeted signals. The self–other opposition collapses; the political Other gains quasi-subjectivity—it sees, speaks, and acts within the patient.

They are after me because I am a Kemalist and I defend the Republic. They follow me to my home, they listen to my phone calls. They want to make an example out of me.

All the articles I wrote online are being tracked. The CIA, FBI, and MIT are working together to eliminate me because they know my ideas are dangerous to their new world order.

#### The foreign other as enemy: global paranoia and psychotic cosmopolitics

This theme places the persecutory subject beyond the national frame—the United States, Russia, Saudi Arabia, Germany—thus expanding the delusional domain to a transnational horizon. The foreign state is presented not merely as an observer, but as an omnipotent actor operating across borders via satellites, secret agents, financial systems, or religious mechanisms. These narratives often arise from a delusional mood—a pervasive feeling that the world has become strange and personally significant —in which global symbols—flags, embassies, headlines—become hyper-significant, and media cues are engaged as direct forms of communication through hyper-reflexivity. The body is experienced as a geopolitical boundary: skin, skull, and internal organs are imagined as domains intervened upon by foreign technologies, penetrated by “signals,” or marked by ideological or religious laws.

As a communist, I am on a list. The Americans will find me, no matter where I go. They will take me to a secret prison and torture me for information.

I saw on TV that our government made a deal with Saudi Arabia. Now they are enforcing their laws here. If I don’t wear a headscarf when I go out, their agents will find me and punish me.

These experiences intertwine geopolitical anxiety with a disturbed sense of self and disturbances in the self–world boundary. Personal identity is encoded as a node in global conflict; one’s life story fuses with Cold War scenarios, post-9/11 security narratives, or transnational culture wars. Temporal structure also shifts: distant events are felt as simultaneous and retaliatory, while prophecy and threat circulate in a cosmopolitan imagination. The foreign power becomes a disembodied but intimately intrusive enemy Other—nowhere and everywhere; nation, ideology, and even gendered embodiment are read as punishable signs. Compared to localized state themes, the persecutor here is radically external, amplifying helplessness through scale and distance, yet evoking personal closeness via technology and global narratives.

#### The leader’s gaze: from symbolic authority to personalized persecution

This theme highlights cases where the persecutor is not a diffuse apparatus but a directly felt, singular, and inevitable named political leader—such as Turgut Özal, Tansu Çiller, or international figures like Bill Clinton. Patients report that these leaders are personally plotting against them. The authority figure is depicted as omniscient and omnipotent; essentially, third-person distance transforms into second-person address. What is normally symbolic is experienced literally: the leader’s gaze penetrates the patient’s world, and public speeches and media images take on a private communicative form.

Tansu Çiller and Bill Clinton are working together. I know they are conspiring against me personally because I discovered their secret plans for the country.

Every time Özal appears on television, he is speaking directly to me. He is threatening me with his gestures, telling me he will have me kidnapped if I don’t cooperate.

Here, the persecutor is not a nation or institution but a charismatic body and voice; media telepresence is experienced as co-existence. Temporal structure often fuses biographical turning points with political cycles (e.g., elections, crises), such that the leader’s career defines the arc of the patient’s life. Within this structure, symbolic authority is no longer a background myth but the founding principle of worldhood: the leader sees, speaks, and wills through the patient, collapsing the ordinary ontological distance that sustains interpersonal reality.

#### Illegitimate pursuers: terrorist groups and nameless powers

This theme involves cases in which the sense of being targeted and followed is experienced through non-state but organized and illegitimate structures. ISIS, the counter-guerrilla, or unnamed “organizations” are described by patients as forces trying to annihilate them.

I can’t stay in one place for too long. ISIS is coming to kill me. They know who I am and they have my photograph.

The counter-guerrilla has marked my house. I saw the signs they left. They believe I am a traitor and they will silence me.

Here, the persecutory power is an invisible but potent underground structure. These narratives often accompany the belief that the patient is “an important person” or “holds a secret,” making them a target. These delusions sit at the intersection of self-referential grandiosity and moral delusions: the patient is a specially chosen figure who also deserves punishment. The source of power is unclear, but it is absolute and relentless.

These narratives presume constant surveillance and tracking over the self; yet this time, the surveillance comes not from the state but from an “invisible” underground network. Environmental uncertainties and random events are interpreted as traces of the organization. Such delusions often coexist with mistrust toward institutional care and healthcare workers, who are perceived as agents of the organization in the patient’s view.

### Category II: The self reconfigured through political Myths

This category gathers themes where the patient’s fracturing sense of self is reorganized and given meaning through identification with powerful political symbols, historical narratives, or collective identities.

#### Grandiosity as a response to fragmentation: historical identification and mythic selves

This theme captures a compensatory reorganization of the self in which patients identify with prominent national or global figures (e.g., founding leaders, prime ministers, military commanders). Grandiosity here is not merely an inflation of self-esteem; it functions structurally as a response to fragmentation: a fragmented sense of self is reassembled through hyper-identification with culturally saturated symbols that promise wholeness, mission, and moral significance. These cases reveal a fusion of personal and historical time—a disturbance of narrative temporality—where individuals not only remember history but experience themselves as living and authoring it. The emerging “mythic self” is often ambivalent rather than triumphant: alongside claims of extraordinary status, patients often describe burdens, responsibilities, and fear of failing a historical mission. This ambivalence shows that grandiosity carries persecutory tones; diffuse anxiety and loss of agency are reorganized into a readable narrative of leadership, sacrifice, and destiny.

People may not recognize me now, but I am Atatürk. I have returned to see the state of the Republic and to save it once again from its enemies.

I was given a secret tape that contains information that will change world politics. It is my duty to deliver it to President Clinton personally. The fate of millions depends on it.

Clinically, focusing only on content (“I am a national leader”) risks missing the underlying structures. In sociocultural contexts where charismatic leadership and national salvation hold central symbolic value, these identifications provide a ready template to reassemble a disintegrating self. In this sense, grandiosity is not merely “excess”; it is a culturally mediated technology of self-repair under conditions of existential collapse.

#### Contaminated selves: the ethno-religious other as ontological threat

This theme includes delusional threats directed by an Other marked ethnically or religiously—such as Jews, Palestinians, or followers of Freud. The threats are often experienced as symbolic cannibalism, bodily poisoning, or spiritual invasion.

The Jews are trying to take over my body. They want to eat my soul so they can control my will.

Freud is not just a historical figure; his followers are a cult. They are putting his medicines in my food to control my mind and make me one of them.

These narratives emerge through the psychotic transposition of cultural prejudices: imaginary figures from social memory are now experienced as magical-destructive agents. This structure indicates the loss of basic trust: the assumed safety toward others and the world is replaced by a threatening, leaking atmosphere that violates boundaries. The self–other boundary collapses; the Other is internalized and becomes an external invader.

Here, the Other is no longer socially different; it is metaphysically contagious. The patient is not merely watched or followed; their “essence” is targeted. This theme concerns the fragmentation of the self along religious or ethnic lines and the configuration of the Other figure as a corrupting agent of the “pure” self. The structural feature of psychosis—disruption of self-boundaries—finds expression here through religious and ethnic meaning systems.

#### Psychotic nationalism: threats to national integrity and self–state mergers

This theme describes cases where personal disintegration is projected onto the national collective. The patient identifies the fragmentation of the nation with the fragmentation of the self. Delusions often take shape with fantastic elements such as “neutron bombs placed under Ankara” or “factories destroyed by triangle-shaped proton bombs.” Here, the nation becomes an extension of the individual; the subject–object divide dissolves between nation and body.

They have a secret plan to divide the country. They planted neutron bombs under Ankara and Istanbul. I am the only one who knows the codes to disable them.

Our enemies are using new technology. They are destroying our strategic factories with invisible, triangle-shaped proton bombs. The economy will collapse, and so will I.

This condition is characterized by the merging of the self with an external collective entity (state, nation, homeland). National borders are no longer external but metaphors of internal boundaries. The existence of the nation reflects the integrity of the self. Delusional collapse is not merely personal; it is symbolized through the disintegration of the national fabric. In these narratives, political identity becomes not just an ideological position but the vehicle of psychotic narrative. Violations of borders (invasion of the country) overlap with internal violations (dissolution of the self).

Temporal structure also typically collapses; the future is saturated with catastrophe, and the past is continually reinterpreted through invasion. A prophetic political tone accompanies the delusions, and the fate of the self becomes inseparable from the fate of the nation. This theme represents the convergence of classic schizophrenic disintegration with nationalist mythology: the psychotic worldview is woven with conspiracy theories threatening national integrity, and this threat becomes inseparable from the patient’s identity.

## Discussion

This study set out to illuminate how political contexts inflect the content of delusions in psychotic disorders by undertaking a reflexive thematic analysis of archived inpatient records spanning 1985–2020 in Turkey. Rather than treating delusions as isolated cognitive anomalies, we examined them as meaning-bearing narratives situated within specific social and historical environments, a stance that accords with a view of the mind as shaped by social relations—echoing Marx’s sixth thesis on Feuerbach that human essence is constituted in, and through, the ensemble of social relations [29]. Taken together, our findings underscore that politically themed delusions are not random; they coalesce around culturally resonant actors, institutions, and events, and they reorganize personal experience in ways that are legible against the background of Turkey’s political history and its global entanglements [5].

A concise summary of the principal findings is warranted before interpretation. Across the corpus, patients recurrently articulated experiences of invasive or persecutory intrusion by state institutions; conflated their personal significance with national or global leadership; understood themselves as targeted because of ideological identity; located threat in foreign state actors; attributed direct agency to named political leaders; recast ethno-religious others as ontological contaminants; fused self-integrity with national integrity; and construed pursuit by non-state, clandestine organizations. These configurations were not uniformly distributed by time or biography; rather, they often echoed moments of political upheaval, regime change, geopolitical tension, and highly mediatized events, producing an overall picture in which political symbols and figures are recruited as building blocks for delusional systems precisely because they are already overrepresented in public discourse and collective memory [19].

The sociohistorical embedding of delusional content is particularly salient in the Turkish context. Turkey’s second half of the twentieth century and the early twenty-first century were punctuated by coups, states of emergency, ideological polarization, and shifting alignments with global powers, and against this background many patients narrated the state—police, military, intelligence services—as omnipresent, invasive, and punitive [30–32]. The long shadow of the 1960, 1971, and 1980 coups, and the attempted coup of 2016, left behind a repertoire of widely shared expectations about surveillance, arrest, and repression; these expectations did not create psychosis, but they furnished a ready-made vocabulary of threat that could be taken up in delusional meaning-making [32]. Our material also registered the enduring salience of the right–left divide that structured social and campus life in the 1960–1970 s, including fears of politically motivated violence and conspiracies attributed to “the other side,” with several patients articulating the conviction that they were singled out for their ideological commitments in ways that mirror how identity positions were historically encoded as public risks rather than merely private stances [32].

The transnational dimension of threat likewise emerged with force. As a NATO country situated at the intersection of Europe and the Middle East, and as a society steeped in Cold War legacies of espionage and proxy conflict, Turkey’s public imagination has long circulated images of foreign interference and technological reach, and it is therefore notable that patients frequently invoked the United States, Russia, Saudi Arabia, and other foreign powers as persecutors, sometimes linked to satellites, clandestine agents, or religious authority. The presence of global political figures—appearing not as abstractions but as direct actors in patients’ lives—speaks to the power of mediatized leadership as a symbolic resource, and highly visible moments such as diplomatic visits or widely covered scandals can amplify the recognizability of certain names and endow them with narrative utility in delusional systems [23]. In parallel, we observed a recurrent fusion of self and nation, wherein national disintegration or foreign invasion is experienced as an attack on bodily integrity and where bombs under cities or threats to factories become analogues for internal rupture; clinically, such fusions suggest that political news and anniversaries of historical events may function as triggers or organizers of distress in vulnerable patients [19].

The appearance of clandestine, non-state persecutors—such as “counter-guerrilla,” ISIS, or unnamed “organizations”—further reflects a broader social vocabulary of hidden networks and deep structures, recapitulating enduring narratives in public life about invisible hands and shadow governance [25]. For many patients, these culturally available narratives provide an account of why things happen and of who, precisely, is responsible, transforming diffuse anxiety into systematized explanation and thereby conferring an uneasy sense of order on otherwise overwhelming experience [25]. Importantly, politically themed delusions are not synonymous with political interest or activism: many patients reporting surveillance or persecution had no documented histories of political engagement, and political content should therefore not be read as evidence of political action but, rather, as an index of how widely available symbolic resources are pressed into service to make sense of distressing phenomena [22, 23].

Grandiosity warrants particular nuance within this matrix. Identification with national leaders or founding figures—most prominently Mustafa Kemal Atatürk—should not be reduced to inflated self-regard; in our records such identifications frequently contained ambivalence (duty, burden, fear of failure) and unfolded as attempts to restore coherence and agency amid disorganization, especially in social worlds where leadership figures are narratively cast as saviors, reformers, or embodiments of national destiny [33]. Psychoanalytic and social-cognitive accounts suggest that such identifications can serve as a defense against feelings of powerlessness, allowing individuals to integrate culturally admired images of authority into a threatened sense of self. The blurring of self–other boundaries in psychosis may further facilitate this process of absorbing public symbols into one’s personal identity [34, 35]. Seen in this light, grandiosity is culturally mediated and, at times, defensive, and an appreciation of its local symbolic scaffolding has implications for engagement, formulation, and risk assessment.

Several plausible pathways link political contexts to the architecture of delusional systems without implying that politics “causes” psychosis. First, political life offers narrative affordances—characters (leaders, agencies), structures (conspiracies, elections, coups), and plots (betrayal, rescue)—that can organize otherwise chaotic experience; when personal meaning is at risk of fragmenting, narratives with clear antagonists and grand stakes may be preferentially recruited [25]. Second, repression and states of emergency cultivate surveillance expectations that, once sedimented in collective memory, can be generalized in personal life and render everyday ambiguity legible as monitoring. Third, conspiracist ideation in public discourse supplies ready-made explanatory frameworks—hidden causes, deep states—that are portable into private experience and, once adopted, can tighten and self-seal, especially where trust is already depleted. Fourth, mediatized leaders and spectacular events multiply salience and familiarity, and familiar names are therefore more likely to be recruited under stress into personal narratives. Fifth, political identities that are publicly consequential—subject to sanction or pride—are more easily experienced as essential properties of the self, and when threat is generalized, identity can feel like a target painted on one’s being. Finally, grandiose identifications can function as compensatory reorganizations when control is diminished, drawing on culturally available images of decisive capacity, while boundary blurring promotes external attribution of agency in ways that make political symbols feel personally addressed [34, 35].

Our findings sit comfortably within a comparative literature that has repeatedly observed tracking between delusional content and socio-political climates. Studies from diverse settings have noted that persecution and grandiosity dominate politically themed delusions and that salient national conflicts—from the Cold War to regime changes—are mirrored in patients’ narratives as those conflicts permeate public consciousness [19, 22, 23]. The present material contributes by specifying how these broad tendencies took shape in Turkey: the repetition of state-centered persecution under the shadow of coups; the salience of founding or charismatic leaders as figures of identification; the appearance of foreign power as persecutor within a NATO-borderlands imagination; and the adoption of clandestine, non-state enemies in line with locally circulating ideas about deep structures. These convergences and divergences suggest that politically themed delusions are at once translatable across contexts (sharing structural features) and irreducibly local (surfacing particular symbols and actors), a conclusion that dovetails with prior work showing that delusional content changes with technological developments, political processes, and sociocultural shifts [5, 11, 13–17, 19].

A theoretical question that arises from these observations concerns whether the gulf between psychotic and non-psychotic political thinking is primarily quantitative rather than qualitative. We caution against overstatement, yet two considerations are notable: in periods of heightened political anxiety, general discourse becomes saturated with motifs—surveillance, betrayal, foreign manipulation—that also commonly populate delusional systems, and the very public figures and institutions that dominate everyday conversation appear within delusional content as well. The difference, then, may lie less in the raw materials than in rigidity, incorrigibility, and personal centrality; put differently, politically themed delusions may extend and intensify shared narrative resources rather than inventing entirely new ones, a view consistent with the claim that delusions are shaped by the social relations and symbolic economies in which minds are embedded [29].

When translated into clinical practice, this contextual frame yields concrete principles that can orient everyday assessment and intervention. Assessment should be deliberately context-sensitive; routine history taking ought to map each patient’s “political horizon of meaning,” identifying which institutions, events, and identities are salient and tracing how these attach to perceived threat or to a sense of “mission,” while acknowledging the historical roots of mistrust without endorsing delusional claims. Risk formulation is strengthened by attention to temporal coupling: where the self is fused with the nation or direct agency is ascribed to powerful leaders, news cycles and anniversaries of historical events may constitute “risk windows,” enabling teams to calibrate monitoring and support accordingly. The therapeutic alliance is consolidated by a stance that is alert to political history and present realities yet remains anchored in proportionality, corroboration, and personal centrality, thereby avoiding the epistemic invalidation that can follow from dismissing political content as mere noise. Psychoeducation and cognitive work can create space for alternative readings of ambiguous cues and for jointly weighing the costs of global, self-referential interpretations; at the same time, cultural formulation and documentation should record not only symptom form but also the cultural specificity of content—named institutions and leaders—and its personal significance, thus strengthening continuity of care and reducing the risk of misinterpretation by new providers. Finally, in contexts where surveillance and repression have in fact occurred, ethical care requires proportionate verification rather than reflexive dismissal of reported threats, keeping in view the rare but meaningful possibility of a non-delusional kernel.

Methodologically, employing reflexive thematic analysis on archived clinical narratives affords the capacity to detect patterns spanning a long time frame and to demonstrate how political symbols recur and are reconfigured; the archive also supplies a diversity of voices and contexts that would be difficult to assemble prospectively. Nonetheless, several limitations must be acknowledged candidly. First, our reliance on clinician-authored records means the data are inherently filtered through the clinician’s perspective, which may have shaped how patients’ experiences were documented. Second, over such a long and politically charged period, we cannot rule out the possibility that clinicians may have omitted or softened certain politically sensitive details from the record, representing a potential bias. Third, a key limitation is the absence of a systematic temporal analysis mapping changes in theme frequency to specific political events. Such an analysis was beyond the scope of this qualitative study due to inconsistencies in archival data quality over four decades, but it remains a crucial area for future investigation. Shifts in diagnostic systems and recording practices over the study period may also have influenced what was written, while translation from Turkish to English—undertaken by bilingual team members with secondary review—inevitably entailed choices that shade meaning. Limitations beyond the archival constraints must be acknowledged candidly. The sample is drawn from a single tertiary center in Turkey, which limits generalizability across regions and health systems, and we did not attempt fine-grained time-locking of delusional content to specific political events, an analysis that would require prospectively dated narratives and linkage to external timelines. We also lacked a comparison group of inpatients with non-political delusions that could sharpen claims about distinctiveness, and we did not capture media consumption or personal political engagement, variables that might moderate how symbols are selected and organized. Finally, while we reported theme frequencies to enhance transparency, our aim was interpretive adequacy rather than prevalence estimation, and the numbers should be read accordingly.

Future research can extend these insights through mixed designs that combine prospective, time-stamped qualitative interviews with ecological data on news cycles and social media attention, thereby enabling analysis of how public events are appropriated into personal narratives in near-real time. Cross-national comparisons anchored in matched historical periods would allow stronger inferences about the cultural specificity of symbol repertoires, while within-country, multi-site studies could examine regional differences in the salience of particular institutions or leaders and the uptake of foreign versus domestic persecutors. Clinically, intervention trials might test whether context-attentive psychoeducation and cognitive strategies reduce distress or rigidity without dismissing political meaning. Furthermore, training curricula for psychiatry residents could incorporate modules on interpreting delusional content in its sociohistorical context, with attention to ethical pitfalls such as re-traumatization through invalidation. Public mental health communication during crises should also be scrutinized for unintended amplification of persecutory narratives and recalibrated toward transparency and reduced ambiguity.

## Conclusions

In conclusion, politically themed delusions in psychotic disorders are not noise at the margins of psychopathology; they are structured narratives that draw on the most available and affectively charged materials in public life. In the Turkish context examined here, state institutions, charismatic leaders, foreign powers, clandestine organizations, and ethno-religious others served as recurrent anchors for meaning, and these anchors were not arbitrarily chosen but reflected decades of political experience, memory, and media representation. Recognizing this does not collapse the distinction between psychotic and non-psychotic thought; rather, it equips clinicians with a better map—one that shows where patients are drawing their symbols from, why these symbols feel inevitable, and how therapeutic work might proceed without either pathologizing politics or ignoring symptom form.

## Data Availability

The datasets analyzed comprise de-identified excerpts from confidential hospital records and are not publicly available due to legal/ethical restrictions and risk of re-identification. De-identified analytic are available from the corresponding author on reasonable request and subject to institutional and ethics approvals.
